# Effective Pollination Period and Parentage Effect on Pollen Tube Growth in Apple

**DOI:** 10.3390/plants10081618

**Published:** 2021-08-06

**Authors:** Stefan Roeder, Sara Serra, Stefano Musacchi

**Affiliations:** 1 Tree Fruit Research and Extension Center, Washington State University, Wenatchee, WA 98801, USA; sara.serra@wsu.edu (S.S.); stefano.musacchi@wsu.edu (S.M.); 2 Department of Horticulture, Washington State University, Pullman, WA 99164, USA

**Keywords:** maternal and paternal effects, in vivo pollen tube growth, *Malus domestica* Borkh

## Abstract

Flower receptivity is a limiting factor for the fertilization of several tree fruit. The effective pollination period (EPP) can be used to determine flower longevity and identify limiting factors by assessing stigmatic receptivity, pollen tube growth rate, and ovule longevity. EPPs were determined for three apple cultivars under natural field conditions in Washington State in 2019 and 2020. In addition, a greenhouse study, performed under semi-controlled conditions, evaluated the influence of six maternal parents on the pollen tube growth performance of six pollen sources. The duration of the stigmatic receptivity ranged from 6.3 to 8.1 days, depending on the cultivar and year—pollen tubes required between 5.5 and 7.0 days from the stigma to reach the ovules. Ovule longevity of non-pollinated flowers varied between 8.2 and 11.3 days. Combinations of these factors resulted in EPPs ranging from 3.0 days for ‘Rubinstar’ to 5.6 days for ‘Olsentwo Gala’ in the present experimental conditions. The greenhouse study revealed that parentage affected pollen tube growth performance. Importantly, a significant interaction between maternal and paternal factors indicated that the performance of different pollen sources depended on the maternal parent and that general recommendations on pollination need to account for the maternal parent.

## 1. Introduction

Pollination and fertilization are two key processes that impact fruit set and quality of apple (*Malus domestica* Borkh.) [1,2,3]. Usually, only 5% to 8% of all flowers must set to achieve economically acceptable yields [4]. However, several plant, pollinator, and environmental factors can act alone or in combination to limit fruit set by reducing pollination and fertilization success during the flowering period.

Most apple cultivars are self-incompatible and require cross-pollination with genetic compatible and viable pollen to fertilize the ovules [5]. Self-incompatibility is caused by a genetic GSI system, which is also exhibited in sweet cherry (*Prunus avium* L.), and pear (*Pyrus communis* L.) [5] and controlled by multi-allelic genes on a single S-locus [6]. Therefore, sufficient cross-pollination with compatible pollen is a key requirement for fertilization [5]. In commercial orchards, integration of appropriately spaced pollinizers and provision of managed pollinators are currently the standard practice to ensure cross-pollination. For apple, the use of crabapples can be an effective option because of their overlapping flowering time and attractiveness to honeybees [7]. Some popular crabapple pollinizers are ‘Mt. Blanc’, ‘Indian Summer’, ‘Chestnut Crab’, ‘Thunderchild’, and ‘Wickson Crab’ [8]. Previous reports investigating the pollen tube growth performance of some crabapple cultivars at different temperatures have already indicated that performance depends on both maternal and paternal effects [8]. In addition, later reports have further highlighted the importance of selecting suitable pollinizers for effective pollen tube growth under natural conditions [9].

Williams [10] introduced the concept of the effective pollination period (EPP) to investigate the effect of summer nitrogen applications on flower quality in the following year. According to Williams [10], the EPP concept includes three main factors: stigmatic receptivity, pollen tube growth rate, and ovule longevity. EPP is calculated as the difference between ovule longevity and the required time for the pollen tube to reach the ovules. An alternative to the ‘indirect method’, which requires the determination of the three components (stigmatic receptivity, pollen tube growth rate, and ovule longevity) based on microscopic observations, is the use of sequential pollination and the evaluation of the initial or final fruit set [11]. This method is often referred to as the ‘direct method’ and includes the emasculation, isolation, and sequential cross-pollination of flowers. While this approach is less time-consuming, it does not provide information about the limiting factors (stigmatic receptivity, pollen tube growth, and ovule longevity). 

A third approach, described by Ughini and Roversi [12], begins by isolating several branches prior to bloom. Isolation bags are then removed in daily intervals, and the number of flowers per shoot as well as the number of open flowers and the final fruit set, are recorded. Based on the results, a fruit set curve can be created, and the EPP can be estimated. The main difference between the Ughini and Roversi [12] method and the sequential pollination of emasculated and isolated flowers is that the pollination event depends on natural orchard conditions, and no emasculation or artificial hand-pollination is involved. Furthermore, the EPP is not based on the longevity of a single flower. Instead, the fruit set is based on flower clusters. Therefore, Ughini and Roversi [12] defined the calculated EPP as a production-permitting period (PPP).

The EPPs were determined for several cultivars of apple [13,14,15,16], apricot (*Prunus armeniaca* L.) [17], pear [13,14,16], sour cherry (*Prunus cerasus* L.) [18], sweet cherry [12], kiwifruit (*Actinidia* ssp.) [19], and olive (*Olea europaea* L.) [20,21]. So far, EPPs for apple cultivars have been reported under controlled conditions for ‘Golden Delicious’ [14], under natural conditions for ‘Cox’s Orange Pippin’ in the United Kingdom [22], as well as for ‘Golden Delicious’, ‘Red Chief Delicious’, and ‘Golden Delicious Tardio’ in Mexico [15]; they have also been reported for 13 older apple cultivars in the United Kingdom [16].

Because of the different climatic conditions during bloom, results from research studies with significantly different weather conditions cannot be easily compared with other growing regions. Therefore, the specific objectives of this study were (1) to determine the effective pollination period by assessing stigmatic receptivity, pollen tube growth rate, and ovule longevity in three apple cultivars under natural orchard conditions in Washington State and (2) to determine potential parentage effect on pollen tube growth rate under semi-controlled greenhouse conditions utilizing six maternal and six paternal apple cultivars. 

## 2. Results

### 2.1. Effective Pollination Period

#### 2.1.1. Stigmatic Receptivity

Stigmatic receptivity was assessed based on pollen adhesion and pollen germination (Table 1). Overall, the stigmas of the evaluated cultivars supported the adhesion of pollen grains from 8.5 (‘Olsentwo Gala’ in 2019 and 2020) to 9.4 days (‘Golden Delicious’ in 2019). The ability to support pollen germination was approximately 1.3 days shorter than the ability to support pollen adhesion, with differences ranging from 0.7 (‘Olsentwo Gala’ in 2020) to 2.2 days (‘Olsentwo Gala’ in 2019).

#### 2.1.2. Pollen Tube Growth Rates

Depending on the cultivar and year, ‘Granny Smith’ pollen tubes reached the ovules between 5.5 days (‘Olsentwo Gala’ in 2020) and 7.0 days (‘Rubinstar’ in 2020) after pollination (Table 2). The average time for the pollen tube to reach the ovules, over all cultivars and years, was 6.3 days. In 2019, pollen from ‘Snowdrift’ and ‘Granny Smith’ was compared utilizing ‘Golden Delicious’ and ‘Olsentwo Gala’ flowers. ‘Snowdrift’ pollen tubes required an additional 0.4 and 0.6 days at the same temperature to reach the ovules in ‘Golden Delicious’ and ‘Olsentwo Gala’, respectively. 

#### 2.1.3. Ovule Longevity

Ovule longevity varied among cultivars and years (Table 3). In 2019, the longevities of ovules in ‘Olsentwo Gala’, ‘Rubinstar’ and ‘Golden Delicious’ were 9.3, 9.8, and 9.8 days, respectively. Slightly longer longevities were observed in 2020; 11.1, 10.0, and 11.3 days in ‘Olsentwo Gala’, ‘Rubinstar’, and ‘Golden Delicious’, respectively. 

#### 2.1.4. Estimated Effective Pollination Period

**Figure 1:**
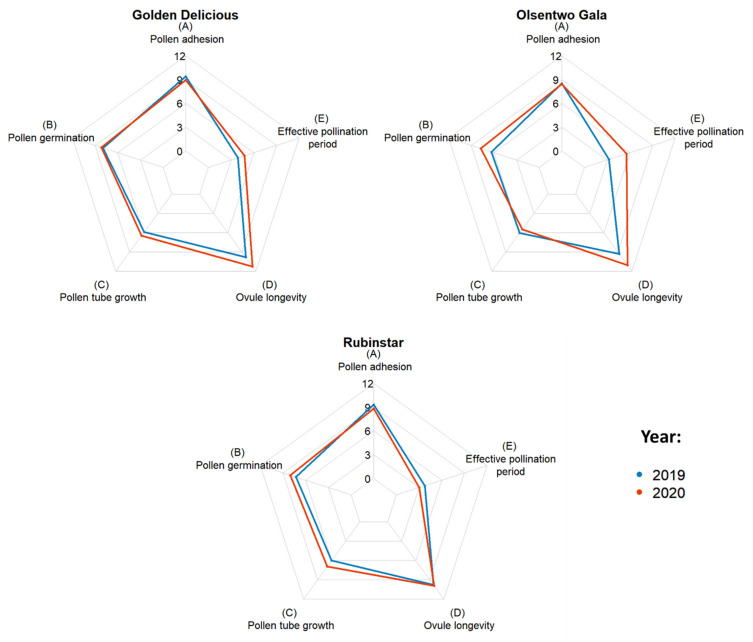
Duration of stigmatic receptivity based on pollen adhesion (**A**), and pollen germination (**B**), pollen tube growth from stigma to ovule (**C**), ovule longevity (**D**) and effective pollination period (**E**) of ‘Golden Delicious’, Olsentwo Gala’, and ‘Rubinstar’ flowers in 2019 (blue) and 2020 (yellow).

EPP was calculated by subtracting the time required for the pollen tube to reach the ovules from the ovule longevity (Table 3). Averaged over both years, ‘Golden Delicious’, ‘Olsentwo Gala’, and ‘Rubinstar’ had EPPs of 4.3, 4.4, and 3.4 days, respectively. In 2020, the EPPs of ‘Golden Delicious’ and ‘Olsentwo Gala’ were 0.9 and 2.3 days longer, respectively, compared to 2019. The EPP of ‘Rubinstar’, which was 3.8 days in 2019, was approximately 0.8 days shorter than in 2020. Figure 1 shows the year-to-year variability of ‘Golden Delicious’, ‘Olsentwo Gala’, and ‘Rubinstar’.

**Figure 2:**
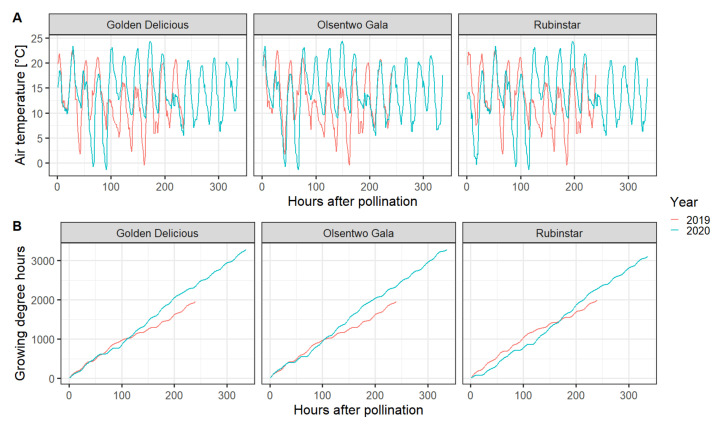
Hourly temperature (**A**) and cumulative growing degree hours with a base temperature of 4.5 °C (**B**) during the experimental period in 2019 (red line) and 2020 (blue line). Data were acquired from the WSU AgWeatherNet website (https://weather.wsu.edu/, accessed on 31 July 2020).

All components of the EPP experiment were performed under field conditions utilizing pollination sleeves to prevent unwanted cross- or self-pollination; however, the sleeves affected several environmental factors, namely temperature, relative humidity, wind speed, and solar radiation. Compared with the environmental conditions of the unbagged flowers (no sleeves), sleeves increased the air temperature by a maximum of 2.2 °C during the afternoon (12:30–16:30). However, the temperature inside the bags was 2.2 °C lower during the early morning (6:00–8:30). Averaged over the whole experimental period, the mean temperature difference was ±0.01 °C. The relative humidity inside the bags was about 2.5% higher than outside the sleeves. Furthermore, the sleeves eliminated wind and reduced the photosynthetic active radiation by approximately 55%. In 2020, all cultivars experienced two nights with temperatures below 0 °C. On 16 April 2020 and 17 April 2020, temperatures reached a minimum of −1 and −1.7 °C, respectively (Figure 2).

### 2.2. Parentage Effect on Pollen Tube Growth

**Figure 3:**
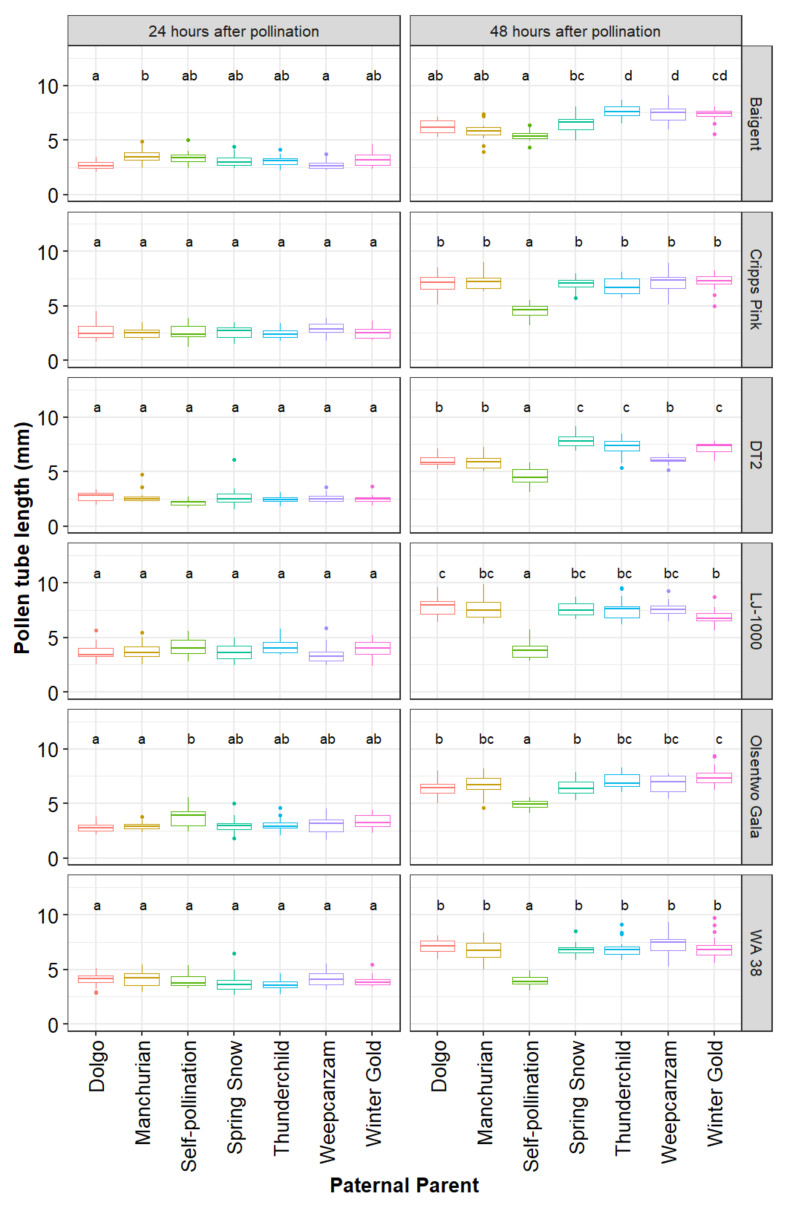
Parentage effects on pollen tube growth. Six maternal parents (‘Baigent’, ‘Cripps Pink’, ‘DT2’, ‘LJ-1000’, ‘Olsentwo Gala’, and ‘WA 38’) were cross-pollinated using pollen from six paternal crabapple parents (‘Dolgo’, ‘Manchurian’, ‘Spring Snow’, ‘Thunderchild’, ‘Weepcanzam’, and ‘Winter Gold’) and a self-pollinated control. Flowers were collected 24 and 48 h after pollination. Different letters indicate significant differences between paternal parents within a time point and maternal parents (Tukey test, α = 0.05).

The average in vitro pollen germination rate, determined prior to the artificial pollination experiment, differed significantly among cultivars (*p* < 0.001). Overall, in vitro germination rates ranged from 50.2% (95% CI: 43.8–57.7%) in ‘Manchurian’ to 78.2% (95% CI: 70.0–87.4%) in ‘Winter Gold’. The three main effects, maternal parent (χ2 = 231.02, df = 5, *p* < 0.001), paternal parent (χ2 = 362.27, df = 6, *p* < 0.001), and time point (χ2 = 6421.79, df = 1, *p* < 0.001), as well as the following interaction terms maternal parent x paternal parent (χ2 = 140.94, df = 30, *p* < 0.001), maternal parent x time point (χ2 = 121.73, df = 5, *p* < 0.001), paternal parent x time point (χ2 = 474.60, df = 6, *p* < 0.001), and maternal parent x paternal parent x time point (χ2 = 148.20, df = 30, *p* < 0.001) were significant. As a result, pollen tube lengths for every paternal parent were analyzed for every maternal parent and time point separately (Figure 3).

At 24 h following pollination, pollen tube lengths did not differ among the seven paternal parents within styles of ‘Cripps Pink’, ‘DT2’, ‘LJ-1000’, and ‘WA 38’ (Figure 3). ‘Baigent’, ‘Dolgo’ (mean = 2.69 mm, 95% CI: 2.30–3.09 mm) and ‘Weepcanzam’ (mean = 2.72, 95% CI: 2.33–3.12 mm) had significantly shorter pollen tubes than ‘Manchurian’ (mean = 3.59, 95% CI: 3.20–3.99 mm). ‘Olsentwo Gala’, ‘Dolgo’ (mean = 2.81 mm, 95% CI: 2.42–3.21 mm) and Manchurian (mean = 2.92, 95% CI: 2.52–3.31 mm) had significantly shorter pollen tubes than the self-pollinated pollination treatment (mean = 3.76 mm, 95% CI: 3.36–4.15 mm). 

**Figure 4:**
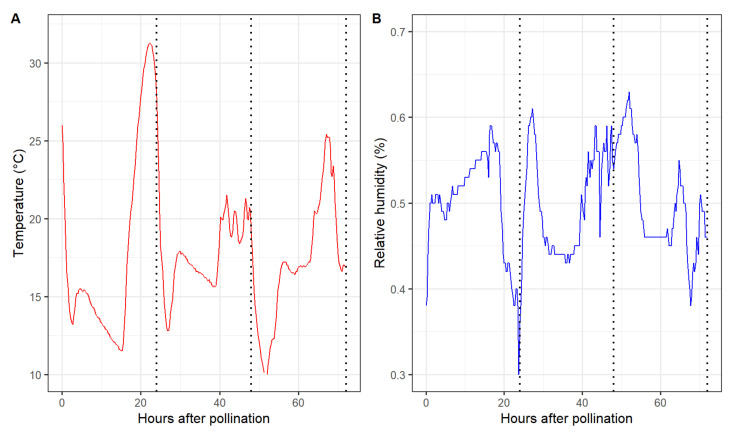
Air temperature (**A**) and relative humidity (**B**) during the experimental period inside the greenhouse. Dotted vertical line represents the sampling time points 24, 48, and 72 h after pollination.

Marked differences in the pollen tube performance among pollen sources were observed 48 h after pollination. Overall, self-pollination resulted in the shortest average pollen tube length in all six maternal cultivars. The pollen tube length of the remaining six pollen parents did not differ in ‘Cripps Pink’ and ‘WA 38’. However, ‘Spring Snow’, ‘Thunderchild’, and ‘Winter Gold’ performed better than ‘Dolgo’, Manchurian’, and ‘Weepcanzam’ in ‘DT2’ styles. All paternal parents, except for the self-pollinated control, passed the base of the styles 72 h after pollination. Therefore, this time point was discarded from further statistical analysis. During the first 24 h, greenhouse temperature values ranged from 11.5 to 31.2 °C, while relative humidity varied between 30% and 59% (Figure 4). Lower temperatures were observed between 24 and 48 h after pollination, with temperature values ranging from 12.8 to 21 °C and relative humidity values between 43% and 60%.

## 3. Discussion

### 3.1. Effective Pollination Period

#### 3.1.1. Stigmatic Receptivity

Stigmatic receptivity based on the ability to support pollen adhesion and pollen germination was not considered a limiting factor because the durations exceeded the estimated EPPs based on the experimentally derived pollen tube growth and ovule longevity. King flowers of ‘Golden Delicious’ had a shorter and more intense stigmatic receptivity when compared to lateral flowers [23]. Up to the present time, no published study has compared the effective pollination period between king and lateral flowers.

In the current study, the stigmatic receptivity was based on the ability of the stigma to support pollen germination using a binary scale (absent-present). Other researchers used quantitative methods to assess stigmatic receptivity, such as the number/percentage of adhered/germinated pollen grains, which provides a more precise representation [24]. However, the sample transfer during processing for imaging led to a loss of pollen grains from the stigmatic surface area. Therefore, pollen grain counts were not made during this study. The trend that pollen germination on the stigmatic surface area decreased more rapidly than pollen adhesion is also in agreement with other studies on apple [24]. Previous reports in sweet cherry (*Prunus avium* L.) and peach (*Prunus persica* L.) have also demonstrated that the timeframe of stigmatic support for pollen tube growth and penetration is shorter than the capacity to support pollen germination, which is, in turn, shorter than the duration of pollen adhesion [25,26]. Temperature can both shorten or extend the period of stigmatic receptivity, depending on the specific range. For example, Prunus avium ‘Summit’ stigmas lost the ability to support pollen germination after eight days, five days, and three days at 10, 20, and 30 °C, respectively [25]. The optimal temperature range for apple stigma to support pollen germination is still unknown. 

#### 3.1.2. Pollen Tube Growth Rate

Pollen tubes reached the ovules between 5.5 and 7.0 days after pollination, which is in accordance with previously published reports under natural field conditions [27,28]. Controlled experiments showed that apple pollen tube growth rates are affected by environmental conditions such as temperature [8]. Therefore, pollen tubes in apple-growing regions with warmer spring temperatures might require less time to reach the ovules. However, higher temperatures have been reported to shorten the longevity of ovules in blueberry [29], sweet cherry [30], and plum [31]. Apple cultivars have different optimum temperatures for maximum pollen tube growth rates. For example, Alvarez et al. [32] showed that, under controlled temperatures, ‘Red Delicious’ pollen reached the maximum growth rate at 22 and 28 °C in ‘Gala’ and ‘Fuji’, respectively. The temperature profile of the 2019 season in the present experiment had a slightly higher minimum (0.9 °C) and lower maximum temperature (22.5 °C) compared to the 2020 season (min. temperature: −1.7 °C, max. temperature: 25.3 °C). The optimum temperature for pollen tube growth may not have been reached for an extended period of time during this experiment. Pollen tube growth rates can be further affected by the cultivar [8]. In 2019, ‘Snowdrift’ pollen tubes required approximately 0.5 days longer to reach ‘Golden Delicious’ and ‘Olsentwo Gala’ ovules than ‘Granny Smith’ pollen. Previous research showed that ‘Snowdrift’ has relatively slow pollen tube growth rates when compared to other crabapples like ‘Evereste’, ‘Indian Summer’, ‘Selkirk’, and ‘Thunderchild’ [6]. Therefore, identifying pollen sources with fast pollen tube growth rates might be an option to extend the effective pollination period by shortening the time between pollination and fertilization.

#### 3.1.3. Ovule Longevity

Ovules of emasculated and unpollinated flowers remained viable for 8.2 to 11.3 days, depending on the cultivar and year. The accumulation of callose has been used as an indicator to determine the ovule senescence in several tree fruit species, such as apple [15], cherry [33], pear [34], and plum [31]. However, Tonutti et al. [35] postulated that ovule fluorescence might be more related to aging of the ovule than the actual viability. The average ovule viability of 63% has previously been reported to be a limiting factor in ‘Golden Delicious’ [15]. However, in the current study, ‘Golden Delicious’ ovules did not show low initial ovule viability within the first 4 days. All investigated ovules of ‘Golden Delicious’ were fully viable (non-fluorescent) for up to 8 and 9 days in 2019 and 2020, respectively. The difference between both studies might be related to different environmental conditions, as Prieto et al. [15] investigated the ovule longevity of ‘Golden Delicious’ under natural conditions in Mexico. However, no final comparison between the present study and Prieto et al. [15] can be made because the environmental conditions were not reported in the latter [15]. In contrast to diploid cultivars, where the egg apparatus matures with the opening of the flower, the egg apparatus of triploid cultivars matures two or three days after anthesis, which results in extended ovule longevity and a longer effective pollination period [28]. However, in 2020, the ovule longevity of the triploid apple cultivar ‘Rubinstar’ was approximately one day shorter than the ovule longevity of the two diploid cultivars ‘Golden Delicious’ and ‘Olsentwo Gala’.

#### 3.1.4. Effective Pollination Period

In this study, EPPs were determined for ‘Golden Delicious’, ‘Olsentwo Gala’, and ‘Rubinstar’ king flowers using the indirect method described by Williams [10]. EPPs for different apple cultivars have been reported to range from two to six days [10], one to nine days [16], and four to 10 days [15]. Here, EPPs ranged from 3.0 days in ‘Rubinstar’ to 5.6 in ‘Olsentwo Gala’. The results suggested that king flowers of ‘Golden Delicious’, ‘Olsentwo Gala’, and ‘Rubinstar’ should be cross-pollinated within 3.0 to 5.6 days after anthesis, depending on the cultivar and year. It should be noted that there was year-to-year variability. The EPP of ‘Olsentwo Gala’, for example, was 40% shorter in 2019 (3.3 days) compared to 2020 (5.6 days). The main reason for the longer EPP in 2020 was the increased ovule longevity (+1.8 days). The specific mechanism for the year-to-year variable is currently unknown. In 2019, heat accumulation expressed as GDH was lower than in 2020. Therefore, ovule longevity was expected to be longer in 2019. The unexpected results could be related to the low temperatures at the beginning of the experiment in 2020, when temperatures reached a minimum of −1 and −1.7 °C on 16 April and 17 April, respectively. However, the exact relationship between temperature and ovule longevity in apple is still currently unknown. Experiments under controlled conditions would be necessary to assess ovule longevities under various temperature regimes. The development of temperature-based models can then help to explain some of the variability between locations or years.

There are several drawbacks when using the ‘indirect method’ described by Williams [8] to estimate EPP. First, Selak et al. [21] determined olive EPP, based on microscopic observations, to be significantly longer when compared to sequential pollination and fruit set data. Therefore, Selak et al. [21] considered the ‘direct method’ more accurate and relevant for growers. Second, the amount of pollen artificially applied to the stigma exceeded the typical natural pollination level. Higher pollen densities have been shown to increase pollen tube growth in Asian pear [36]. It is unknown if higher pollen densities would extend the EPP by shortening the time it takes for the pollen tube to reach and fertilize the ovules. Third, the emasculation process can interfere with natural flower development. Emasculation of flowers was a necessary step to prevent uncontrolled self-pollination; however, research on Japanese plum has shown that flower emasculation can lead to the premature degeneration of ovules [37]. Accelerated ovule degeneration was also observed in emasculated sweet cherry flowers [38]. With regard to apples, flower emasculation has been shown to decrease fruit set but not seed set [39]. This is probably due to the increased biosynthesis of ethylene due to wounding. However, the exact mechanism is currently unknown. 

Another criticism of EPP studies might be the limited comparability across different research studies. Currently, most studies use days as a unit when reporting and comparing EPPs across cultivars. The use of heat accumulation units, such as growing degree days (GDDs), would be more appropriate. GDDs are frequently used in entomological and plant phenological studies to predict development and make seasonal comparisons [40]. However, despite the acceptance of GDDs, this unit has not been implemented in studies that investigate effective pollination periods. Therefore, the effective pollination periods in the present study were also reported in GDDs with a base temperature of 4.5 °C to allow a better comparison with future studies.

### 3.2. Parentage Effect on Pollen Tube Growth 

Previous reports have already described the effect of the maternal and paternal parents on pollen tube growth. For example, DeLong et al. [8] found a significant three-way interaction between three maternal parents, five paternal parents, and four temperatures, indicating complex relationships. In contrast to DeLong et al. [8], where the experimental plants were held in growth chambers under constant temperatures and humidity conditions, the present study was performed under semi-controlled greenhouse conditions. No generalized conclusion can be made for pollinizer performance because of the significant three-way interaction between maternal parent, paternal parent, and sampling time point. Overall, only minor differences between all tested crabapple cultivars were observed 24 and 48 h after pollination, and all pollen tubes reached the base of the style within 72 h. Jahed and Hirst [9] investigated pollen tube growth of four pollen sources (‘Delicious’, ‘Golden Delicious’, *M*. × ‘Ralph Shay’ and *Malus floribunda*) on three maternal cultivars (‘Fuji’, ‘Gala’, and ‘Honeycrisp’) under field conditions and also observed a significant interaction between maternal and paternal parents. In the current study, the *S*-genotype of all maternal parents including ‘Baigent’ (S_2_S_5_), ‘Cripps Pink’ (S_2_S2_3_), ‘DT2’ (S_1_S_9_), ‘Olsentwo Gala’ (S_2_S_5_), ‘LJ-1000’ (S_2_S2_4_), and ‘WA 38’ (S_5_S_24_) are known [41,42]. However, the *S*-genotype of several of the utilized crabapples is unknown, except for ‘Dolgo’ (S_32_S_33_) [42] and ‘Manchurian’ (S_5_S_39b_) [43]. Considering that most of the paternal parents performed significantly better than the self-pollination control after 48 h, the exception observed in ‘Baigent’ styles suggested that all maternal and paternal combinations are at least partially compatible. However, no conclusion can be drawn whether semi-compatible combinations perform differently from fully compatible combinations because of the unidentified *S*-genotype of several paternal parents. Furthermore, it is questionable if artificial hand-pollination studies can be used to study the effects of different cross-compatibility levels. Schneider et al. [44], for example, found no differences between the fruit set of different partially and fully compatible combinations. The authors concluded that the excessive amount of applied pollen might be responsible for non-significant differences. Thus, semi-compatible combinations might only result in the lower fruit set under suboptimal pollination conditions [44]. 

Alvarez et al. [32] used a model-based approach to investigate the pollen tube growth of ‘Red Delicious’ (S_9_S_28_) pollen on ‘Gala’ (S_2_S_5_) and ‘Fuji’ (S_1_S_9_) flowers. Their model showed higher growth rates in the fully compatible combination (‘Gala’ pollinated with ‘Red Delicious’) when compared to the semi-compatible combination (‘Fuji’ pollinated with ‘Red Delicious’) at low temperatures. The authors concluded that this might be attributed to the different compatibility levels. Furthermore, it is currently unknown if pollination studies that use a single pollen donor reflect the natural pollination process. Kron and Husband [45] reported that an increased number of pollen donors might promote seed sets and reduce seed abortion. Currently, most research studies evaluate a single pollen donor at a time.

## 4. Materials and Methods

### 4.1. Effective Pollination Period

The EPP for ‘Golden Delicious’ on ‘M.9’, ‘Olsentwo Gala’ on ‘M9-RN29’, and ‘Rubinstar’ on ‘M.26’ were determined in 2019 and 2020. The trees were planted in alternating rows in 2007 at the Sunrise Research Orchard (Rock Island, WA, USA). EPP was determined by investigating stigmatic receptivity, pollen tube growth rate, and ovule longevity using a microscopic fluorescence approach. The experiments were performed on different days because of the different blooming times among these cultivars (Table 4). The duration of this experiment was 10 days in 2019 and 14 days in 2020. The extended period in 2020 was due to the long ovule longevities observed in 2019. All components of the EPP were assessed on king flowers. In apple, the king flower is the central or apical flower within a flower cluster surrounded by lateral flowers.

#### 4.1.1. Stigmatic Receptivity 

Stigmatic receptivity was determined by emasculating and isolating 60 king flowers in 2019 and 84 king flowers in 2020 for every cultivar. Unopened flowers at the balloon stage were isolated using particle protective sleeves (KleenGuard^TM^ A20, Kimberly-Clark, Irving, TX, USA), and a subset of six flowers per cultivar was cross-pollinated with ‘Granny Smith’ pollen at 24 h intervals. ‘Granny Smith’ pollen was obtained by collecting flowers from the same experimental orchard. The anthers were separated using fine tweezers and air-dried for 48 h at 25 °C. The pollinated flowers were fixed in chilled FAA (46.3% ethyl alcohol, 4.5% formaldehyde, 3.5% methyl alcohol, 2.5% acetic acid; Aldon Corporation, Avon, NY, USA) and stored at 4 °C until further analysis. Prior to the analysis, the FAA solution was gently removed, and samples were rinsed twice for 15 min in 50% ethanol followed by one 15 min rinse in distilled water. Afterward, the samples were transferred to an 8 M sodium hydroxide (VWR International, PA, USA) softening solution. After 16 h, samples were transferred to a 5% potassium hydroxide (VWR International, Monroville, PA, USA) clearing solution for four days. The tissue clearing was used as an additional step to improve the contrast of the pollen tubes and maternal tissue. Next, samples were transferred to a staining solution containing 0.1% aniline blue (Acros Organics, Geel, Belgium) in 0.1 M dipotassium phosphate (pH 10; VWR, Monroville, PA, USA) and dark incubated for 24 h at 25 °C. Styles were then dissected from the pistil at the base of the style and placed on a microscope slide. A drop of fresh staining solution was added onto the slide, and the sample was covered with a coverslip. Pollen adhesion (presence of pollen grains) and pollen germination (presence of germinated pollen tubes) were rated on a binary absence/presence scale. Samples were viewed under an epifluorescence microscope (FM690 TC, AmScope, Irvine, CA, USA). The microscope was equipped with a UV filter cube (excitation bandwidth: 330–385 nm; dichroic longpass mirror: 400 nm; emission filter: 420 nm) and a 100-watt ultra-high pressure mercury lamp.

#### 4.1.2. Pollen Tube Growth Rate

Pollen tube growth rates were determined by emasculating and cross-pollinating, all in the same day, 60 king flowers in 2019 and 84 king flowers in 2020 with ‘Granny Smith’ pollen. In addition, ‘Snowdrift’ pollen was used on ‘Golden Delicious’ and ‘Gala’ flowers in 2019. The flowers were isolated using particle protective sleeves (KleenGuard^TM^ A20, Kimberly-Clark, Irving, TX, USA). Six flowers were sampled daily and fixed in chilled FAA (46.3% ethyl alcohol, 4.5% formaldehyde, 3.5% methyl alcohol, 2.5% acetic acid; Aldon Corporation, Avon, NY, USA) and stored at 4 °C until further analysis. Samples were rinsed with DI water, softened with sodium hydroxide, cleared with potassium hydroxide, and stained with aniline blue following the steps outlined above (4.1.1 Stigmatic receptivity). Prior to microscopic observation, all sepals were removed, and one longitudinal section was made through the pistil using a scalpel. All samples were evaluated on a binary scale, which was based on whether the pollen tubes reached the ovules (0 = no, 1 = yes).

#### 4.1.3. Ovule Longevity

Ovule longevity was assessed by emasculating and isolating, with particle protective sleeves (KleenGuard^TM^ A20, Kimberly-Clark, Irving, TX, USA), 60 king flowers in 2019 and 84 king flowers in 2020 per cultivar. Six flowers were sampled at 24 h intervals and immediately fixed in chilled FAA (46.3% ethyl alcohol, 4.5% formaldehyde, 3.5% methyl alcohol, 2.5% acetic acid; Aldon Corporation, Avon, NY, USA). All samples were stored at 4 °C until further analysis. On the day of the ovule extraction, the FAA solution was removed, and flowers were rinsed three times with distilled water. Afterward, flowers were dissected, and ovules were extracted and transferred into 2 mL microcentrifuge tubes containing 1 mL of 5% potassium hydroxide solution. The ovules were cleared in the dark for 5 days at 25 °C. Subsequently, the clearing solution was replaced with 1 mL of 0.1% aniline blue (Acros Organics, Geel, Belgium) in 0.1 M dipotassium phosphate (pH 10; VWR, PA, USA). The samples were incubated in the dark for 16 h, and six ovules per flower were evaluated on a binary scale (0 = non fluorescent, 1 = fluorescent). Fluorescent ovules are considered non-viable. Ovule fluorescence is caused by callose deposition and has previously been used as an indicator for determining ovule senescence in apple [15].

#### 4.1.4. Climatic Conditions

Hourly air temperature and humidity data during the experimental periods were acquired from the WSU AgWeatherNet website (https://weather.wsu.edu/, accessed on 31 July 2020). Growing degree hours (GDH) were calculated by subtracting a base temperature (4.5 °C) [46] from the hourly average air temperature. Negative GDH was set to 0 before the summation of all values during the experimental period. In addition, Bluetooth^®^-equipped temperature and humidity sensors (Govee, Los Angeles, CA, USA) were used to compare the environmental conditions inside and outside the pollination sleeves. An anemometer (HP-866B-APP, Zhuhai Holdpeak instrument, China) was used to evaluate the effect of the pollination sleeve on the wind speed. Measurements were taken at three time points with wind speeds of 2.9 m s^−1^ (24 April 2020), 4.0 m s^−1^ (18 April 2020), and 5.1 m s^−1^ (23 April 2020). The anemometer was covered with a pollination sleeve after taking the reading under natural conditions. Photosynthetic photon flux density was measured inside the bags at two time points using a quantum sensor (Q53292, Li-Cor Biotechnology, Lincoln, NE, USA) that was attached to a light sensor datalogger (LI-1500, Li-Cor Biotechnology, Lincoln, NE, USA).

### 4.2. Parentage Effect on Pollen Tube Growth

A greenhouse experiment with six maternal domestic and six paternal crabapple cultivars was carried out in 2020 to assess potential maternal and paternal effects on pollen tube growth. Six domestic apple cultivars (‘DT2’/’Pajam 2’, ‘Baigent’/’Pajam 2’, ‘Cripps Pink’/’G.11’, ‘LJ-1000’/’G.41’, ‘Olsentwo Gala’/’Bud.9’, ‘WA 38’/’G.41’) were potted into 38 L containers in April 2018 using commercial potting soil (Sunshine Mix #1, Sun Gro Horticulture, MA, USA). Six crabapple cultivars, including ‘Dolgo’, ‘Manchurian’, ‘Spring Snow’, ‘Thunderchild’, ‘Weepcanzam’ and ‘Winter Gold’ on ‘G.41’ rootstocks were potted into 7.5 L pots using the same potting soil. All plants were fertilized during spring 2018, 2019, and prior to the greenhouse experiment using slow-release fertilizer (Osmocote Plus, 6M, 45 g per pot, Scotts, OH, USA). All domestic cultivars were transferred from outdoor conditions into the greenhouse on 1 January 2020 after receiving approximately 1712 chilling hours (CH, base temperature 7 °C) and on 8 January 2020 after receiving approximately 1833 CH. The crabapples were transferred in three consecutive sets on 25 December 2019 (1557 CH), 01 January 2020 (1712 CH), and 08 January 2020 (1850 CH); sequential transfer was necessary to assure an overlap of the late balloon stage flowers of all cultivars. Flowers from all cultivars were harvested at a late balloon stage on 09 February 2020. The petals were removed, and anthers were separated using fine tweezers and collected in 5 mL centrifuge tubes. Afterward, the anthers were dried for approximately 48 h at 25.0 ± 1.0 °C and relative humidity of 24.4 ± 5.3%. The day prior to the pollination (10 February 2020), 144 flower clusters per cultivar were marked and singled out by removing all flowers within a cluster except for one unopened lateral flower at a late balloon stage. All lateral flowers were depetaled and emasculated the following day using a wire stripper (Cat. No. 11054, Klein Tools, TX, USA). Afterward, 8 sets of 18 flowers from every maternal parent were artificially cross-pollinated with all six crabapple cultivars, a self-pollinated control, and an unpollinated control. Six flowers per treatment combination were harvested in 24 h intervals for up to three days. The sampling protocol was similar to the method described above (see stigmatic receptivity 2.1.1). Styles were observed under a laser scanning confocal microscope (Leica TCS SP-5, Bannockburn, IL, USA) at the WSU Franceschi Microscopy and Imaging Center (Pullman, WA, USA). The samples were excited using a 405 nm diode, and the detector range was set from 420 to 550 nm. The xy-coordinates of the pollen grain and the end of the longest pollen tube were recorded, and the Euclidean distance was calculated. Pollen tube length at the third time point (72 h after pollination) already passed the base of the maternal style. Therefore, only time points one (24 h after pollination) and two (48 h after pollination) were reported. A small amount of dried pollen was suspended in liquid pollen germination media (100 g L^−1^ sucrose, 25 mg L^−1^ boric acid, distilled water, pH 6.1). Afterward, 200 µL of the pollen suspension was transferred onto each of the four compartments of the partitioned 1% agar petri dish. Samples were incubated for 4 h at 25 °C. Pollen germination rate was determined by evaluating at least 300 pollen grains for every one of the four replications. In vitro pollen germination rates were determined by viewing the samples under an inverted light microscope (Leica DMi1, IL, USA). Temperature and humidity data inside the greenhouse were recorded in 15 min intervals using an ATMOS 14 temperature and relative humidity sensor and an EM50 data logger (Meter Group, WA, USA). In vitro pollen germination rates were determined prior to the in vivo pollination. In vitro pollen germination rates of all samples were above 50%.

### 4.3. Statistical Analysis

Statistical analyses were conducted using R [47]. Logistic regression models were fitted using the glm base function. The duration of stigmatic receptivity, pollen tube growth rates, and ovule longevity were determined by calculating the time point where 50% of the samples lost the ability to support pollen germination, reached the ovules and showed senescent ovules, respectively. The EPP in days was calculated by subtracting the ovule longevity from the time required for pollen tubes to reach the ovules. Pollen tube lengths were analyzed by fitting a linear mixed model using the paternal parent, maternal parent, and time point as fixed effect and the flower as a random effect. Linear mixed models were fitted using the nlme packages [48]. The car package [49] was used to perform a type III analysis of variance, while mean separations were performed using the emmeans package [50] and the cld function in multcomp [51]. The Tukey method was used for *p* value adjustment with a significance level of 0.05. All in vivo pollen tube length data (4.2 Parentage effects on pollen tube growth) are reported as estimated marginal means and a 95% confidence interval (95% CI).

## 5. Conclusions

The effective pollination period showed high variability between cultivars and years. Cultivars with a short effective pollination period can exhibit fruit set levels below the recommended crop load level under limited pollination conditions. Even though the determination of the effective pollination period based on fluorescence microscopy has several limitations, here useful information about limiting factors which are pollen tube growth and ovule longevity were provided. While our experiments were performed in Wenatchee (WA, USA), these findings should be useful for different locations with similar climatic conditions. Future research should determine stigmatic receptivity and ovule longevity under controlled temperatures in order to create temperature-based models.

## Figures and Tables

**Table 1:** Duration of stigmatic receptivity based on pollen adhesion and pollen germination of ‘Golden Delicious’, ‘Olsentwo Gala’, and ‘Rubinstar’ flowers in 2019 and 2020.

Year	Cultivar	Stigmatic Receptivity			
		Pollen Adhesion		Pollen Germination	
		Days	GDH ^1^	Days	GDH ^1^
2019	Golden Delicious	9.4	1856	7.9	1484
	Olsentwo Gala	8.5	1656	6.3	1267
	Rubinstar	9.3	1874	7.3	1531
2020	Golden Delicious	9.0	2178	8.2	1998
	Olsentwo Gala	8.5	2057	7.7	1923
	Rubinstar	8.8	1977	8.1	1755

^1^ Growing degree hours (base temperature: 4.5 °C).

**Table 2:** Duration of pollen tubes to reach the ovules of ‘Golden Delicious’, ‘Olsentwo Gala’, and ‘Rubinstar’ flowers in 2019 and 2020.

Year	Cultivar	Pollen Tube Growth	
		Days	GDH ^1^
2019	Golden Delicious	5.9	1175
	Olsentwo Gala	6.1	1214
	Rubinstar	6.0	1328
2020	Golden Delicious	6.5	1534
	Olsentwo Gala	5.5	1318
	Rubinstar	7.0	1435

^1^ Growing degree hours (base temperature: 4.5 °C).

**Table 3:** Duration of ovule longevity and effective pollination period of ‘Golden Delicious’, ‘Olsentwo Gala’, and ‘Rubinstar’ flowers in 2019 and 2020.

Year	Cultivar	Ovule Longevity		Effective Pollination Period	
		Days	GDH ^1^	Days	GDH ^1^
2019	Golden Delicious	9.8	1918	3.9	921
	Olsentwo Gala	9.3	1847	3.3	804
	Rubinstar	9.8	1943	3.8	900
2020	Golden Delicious	11.3	2660	4.8	1042
	Olsentwo Gala	11.1	2606	5.6	1334
	Rubinstar	10.0	2274	3.0	600

^1^ Growing degree hours (base temperature: 4.5 °C).

**Table 4:** Overview of the utilized cultivars, rootstocks, start and end dates of the effective pollination period (EPP) experiment in 2019 and 2020.

Year	Cultivar	Rootstock	Year Planted	Start Date	End Date	Experiment Duration (Days)
2019	Golden Delicious	M.9	2007	23 April	3 May	10
	Olsentwo Gala	M.9-RN29	2007	23 April	3 May	10
	Rubinstar	M.26	2007	22 April	2 May	10
2020	Golden Delicious	M.9	2007	13 April	27 April	14
	Olsentwo Gala	M.9-RN29	2007	14 April	28 April	14
	Rubinstar	M.26	2007	12 April	26 April	14

## Data Availability

All raw or derived data supporting the findings of this study are available from the corresponding author, upon request.
